# Interfacial Built-In Electric Field-Driven Direct Current Generator Based on Dynamic Silicon Homojunction

**DOI:** 10.34133/2020/5714754

**Published:** 2020-06-16

**Authors:** Yanghua Lu, Qiuyue Gao, Xutao Yu, Haonan Zheng, Runjiang Shen, Zhenzhen Hao, Yanfei Yan, Panpan Zhang, Yu Wen, Guiting Yang, Shisheng Lin

**Affiliations:** ^1^College of Microelectronics, College of Information Science and Electronic Engineering, Zhejiang University, Hangzhou 310027, China; ^2^Wuxi Branch of Jiangsu Province Special Equipment Safety Supervision and Inspection Institute, Wuxi 214071, China; ^3^State Key Laboratory of Space Power Technology, Shanghai Institute of Space Power Sources, Shanghai 200245, China; ^4^State Key Laboratory of Modern Optical Instrumentation, Zhejiang University, Hangzhou 310027, China

## Abstract

Searching for light and miniaturized functional device structures for sustainable energy gathering from the environment is the focus of energy society with the development of the internet of things. The proposal of a dynamic heterojunction-based direct current generator builds up new platforms for developing in situ energy. However, the requirement of different semiconductors in dynamic heterojunction is too complex to wide applications, generating energy loss for crystal structure mismatch. Herein, dynamic homojunction generators are explored, with the same semiconductor and majority carrier type. Systematic experiments reveal that the majority of carrier directional separation originates from the breaking symmetry between carrier distribution, leading to the rebounding effect of carriers by the interfacial electric field. Strikingly, NN Si homojunction with different Fermi levels can also output the electricity with higher current density than PP/PN homojunction, attributing to higher carrier mobility. The current density is as high as 214.0 A/m^2^, and internal impedance is as low as 3.6 k*Ω*, matching well with the impedance of electron components. Furthermore, the N-i-N structure is explored, whose output voltage can be further improved to 1.3 V in the case of the N-Si/Al_2_O_3_/N-Si structure, attributing to the enhanced interfacial barrier. This approach provides a simple and feasible way of converting low-frequency disordered mechanical motion into electricity.

## 1. Introduction

With the increasing energy demand and scientific development of human being society, constant efforts have been devoted to maintaining the huge energy consumption while minimizing the earth resource cost [1–4]. In particular, under the development of internet of things [5], there are numerous sensors that should be powered, which are severely required for a self-powered in situ energy [6]. Solar energy can be harvested through separating the photogenerated carriers through the built-in electric field in static heterojunction or homojunction [7, 8]. However, solar energy is not available everywhere and everytime, which limits its application in some fields [9]. Instead, generators harvesting electricity from the environment, especially for widely available low-frequency mechanical energy, could be a feasible way for in situ powering those widely spread sensors for the internet of things [10–13]. In particular, a piezoelectric and triboelectric nanogenerator with alternating output has received many attentions [14–18], however, limited by the high impedance and external rectifying circuit [19–23]. With the rapid development of wearable devices and intelligent monitoring equipment [24–28], it is urgent need to look for a high current density in situ energy generator with matching internal impedance with electron component, as a potential candidate for a light and miniaturized functional device, which overcomes the limitation of the environment and weighty external circuit [29–34].

Traditionally, as a fundamental unit of integrated circuit, the heterojunction or homojunction always attaches extensive concern in the information scientific community, which brings many basic applications and builds up the foundations of the modern information society [35–37]. Recently, a series of efforts in energy society based on the semiconductor system have been achieved to convert mechanical energy into direct current electricity and build up a new platform for developing the new energy society, including the dynamic Schottky diode and PN heterojunction proposed by our group [29–34], the triboelectric nanogenerator proposed by Wang et al. [38–40], the tribotunneling generator proposed by Liu et al. [41–46], the conducting polymer-based direct current generator proposed by Shao et al. [47–50], and others [51–54]. In particular, we have established a self-consistent theory of semiconductor devices, which requires further exploration and deepening. And the requirement of different semiconductors in dynamic heterojunction is too complex to wide applications, generating energy loss for crystal structure mismatch. Hence, a dynamic homojunction structure with the same kind of semiconductor and majority carrier type should be thoroughly explored. Strikingly, the dynamic NN homojunction with different Fermi levels should output higher current density as its higher carrier mobility than PN or PP homojunction, in which only majority carrier transport is involved in electricity generation.

Herein, we propose a high current density dynamic NN Si homojunction-based direct current generator, which can continuously harvest energy from mechanical movement. It is inspiring that these novel generators behave with continuous, high density, and direct current output characteristics, which shows great superiority and potential. The current output of this dynamic NN homojunction generator is derived from the breaking symmetry of depletion region majority carrier distribution, leading to the rebounding effect of the space majority carriers driven by the interfacial built-in electric field (*E*). A dynamic NN Si homojunction generator with short-circuit current density (*J*_sc_) of 214.0 A/m^2^, open-circuit voltage (*V*_oc_) of 0.35 V, and power density of 33.6 W/m^2^ has been achieved. It is noteworthy that the current density of the dynamic homojunction is more than 10^3^-10^4^ times higher than triboelectric and piezoelectric nanogenerators [55–57]. Moreover, compared with the impedance of polymer material-based nanogenerators (~M*Ω*), the internal impedance of this semiconductor homojunction-based direct current generator is rather low (~k*Ω*), which is matching with the impedance of the semiconductor-based information electronic device (~k*Ω*) [58]. Furthermore, we find that the semiconductor-insulator-semiconductor structure-based dynamic P-i-N junction generator outputs higher voltage as high as 1.3 V attributed to the enhanced interfacial barrier, in the case of the N-Si/Al_2_O_3_/N-Si structure [59–63]. Compared with piezoelectric and triboelectric nanogenerators, this dynamic NN homojunction generator with ultrahigh current density can effectively output a direct current without a rectification circuit and storage unit. This dynamic homojunction generator can charge a capacitor without external rectifying circuits quickly, indicating its advantage and potential applications in in situ energy acquisition fields. This approach provides a simple and feasible way of converting low-frequency disordered mechanical motion into electricity, especially the biomechanical energy, wind power, and tidal energy.

## 2. Results and Discussion

**Figure 1:**
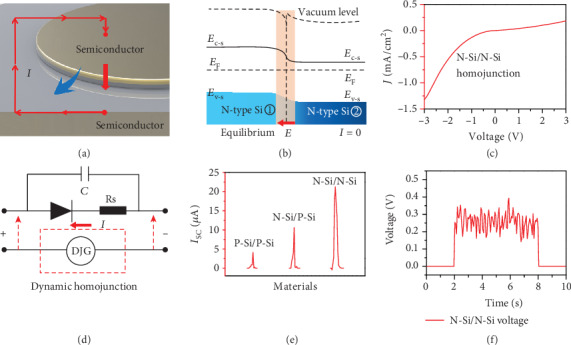
Experimental designs and results of the dynamic homojunction generator. (a) The schematic structure and 3D diagram of the dynamic semiconductor junction-based generator. (b) The band diagram of the static silicon NN homojunction. (c) The rectification characteristic of N-Si/N-Si homojunction. (d) The circuit diagram of dynamic N-Si/N-Si homojunction. (e) The current output of dynamic P-Si/P-Si, P-Si/N-Si, and N-Si/N-Si homojunction generators under the pulse movement mode with a 5.0 N force and a speed of 10.0 cm/s. (f) The voltage output of dynamic NN Si homojunction generator under the continuous movement mode.

Figure 1(a) shows the schematic structure and 3D diagram of the dynamic semiconductor-based generator; the same kind of semiconductor wafers with different Fermi levels is fitted closely to build the depletion region and built-in electric field in the interface. As shown in Figure 1(b), different N-type Si wafers are contacted and a built-in electric field is normally formed in the interface under the equilibrium state. As a result, an ideal rectification characteristic with limited leakage under the negative bias voltage is achieved, which is equivalent to a charging process of the space carriers and forming a symmetry state in the depletion region, as shown in Figure 1(c). The circuit diagram of our dynamic NN Si homojunction is shown in Figure 1(d), which consisted of a diode, an internal resistance *R*_s_, and a junction capacitor. The parallel contact sliding between the two Si wafers can break the static symmetry of homojunction capacitor carriers and generate an electrical output. Primarily, the PP, PN, and NN Si homojunction was explored as a generator with a moving speed of 10.0 cm/s and under a constant 5.0 N force. It can be seen that a pulsed voltage/current is generated, which is caused by the pulse movement signal with an inevitable acceleration and deceleration process. As shown in Figure 1(e), the short-circuit current (*I*_sc_) up to 4.1/10.6/21.4 *μ*A was achieved, respectively, among which the NN homojunction behaves the highest current output, because of the higher carrier mobility than the PN or PP homojunction (the detailed performance of dynamic PN homojunction is shown in Figures S1 and S2). The contact area of the dynamic homojunction in this work is always 0.1 mm^2^ (0.5 mm × 0.2 mm). Accordingly, *J*_sc_ can be calculated to be 41.0/106.0/214.0 A/m^2^ for the dynamic PP/PN/NN Si homojunction, which is more than 10^3^-10^4^ times higher than triboelectric and piezoelectric nanogenerators [55–57]. Only majority carrier transport is involved in the electricity generating process. When the mechanical movement is rapid and continual, the direct voltage as high as 0.3 V can be generated constantly (Figure 1(f)). The interfacial built-in electric field between the NN Si homojunction will bind back the space charges in the depletion region. Diffusion electrons and holes in dynamic homojunction can be directionally separated by the built-in electronic field at the interface, which will generate the continuous current output under the effect of the ultrahigh built-in electric field, allowing for harvesting of energy from mechanical movement continuously. The electricity generation between the same kinds of Si with different Fermi levels indicates that the triboelectric effect is not the key role in the semiconductor-based dynamic junction direct current generator, while the physical picture of rebounding carriers under the interfacial *E* is self-consistent.

**Figure 2:**
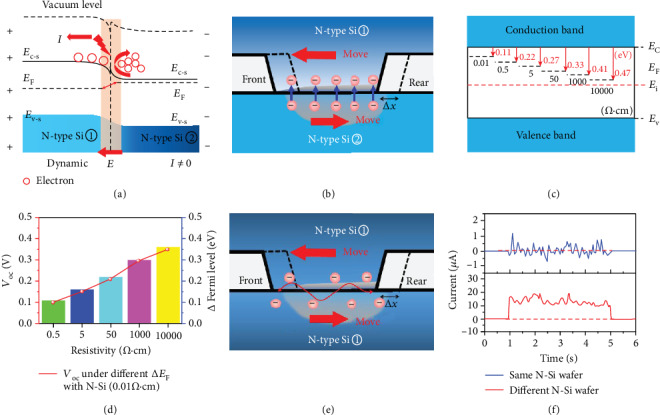
The physical mechanism based on dynamic homojunction under the built-in electric field. (a) The band diagram and electron transport process of the dynamic N-Si/N-Si homojunction generator. (b) The schematic diagram of the dynamic N-Si/N-Si homojunction generator with different Fermi levels. (c) The one-dimensional band alignment of the Fermi level of N-Si substrate with different resistivity of 0.01, 0.5, 5, 50, 1000, and 10000 *Ω*·cm. (d) The relationship between the voltage output and the Fermi level difference between two N-Si substrates. (e) The schematic diagram of the dynamic N-Si/N-Si homojunction generator with the same Fermi level. (f) The current response of dynamic N-Si/N-Si homojunction generator under the continuous movement mode with the same or different Fermi levels.

As shown in Figure 2(a), this mechanical movement process reveals a physical picture of a dynamic homojunction with the rebounding hot carriers, which is very rarely being studied as yet and interesting for further research. Under the Fermi level difference of two semiconductors, the diffusion electrons and holes will transfer into the two semiconductors, respectively, forming a depletion region and built-in electric field, which works as a space carrier charging process of the homojunction capacitor [64, 65]. With the movement of the dynamic homojunction, the rebounding effect of the carriers in the interface will narrow down the space charge region, which works as a homojunction capacitance discharging process, as shown in Figure 2(b). The interfacial *E* plays a key role in the electrical output of the generator, which is related to the Fermi level difference. So, the dynamic N-Si/N-Si homojunction generators with different Fermi levels are explored by using N-Si wafer with different resistivity of 0.01/0.5/5/50/1000/10000 *Ω*·cm.

The Fermi level of the N-Si semiconductor can be calculated by the formula [66]

(1) $$\begin{array}{c} E_{\text{F}-\text{N}}\approx E_{\text{i}}+k_{\text{B}}T\;\ln\frac{N_{D}-N_{A}}{n_{\text{i}}}, \end{array}$$

(2) $$\begin{array}{c} \sigma =\frac{1}{\rho }\approx qn\mu_{n}, \end{array}$$

where *E*_i_ is the middle value of the bandgap; *k*_B_ is the Boltzmann constant; *T* is the temperature; *n* is the electron concentration; *n*_i_ is the intrinsic electron concentration of the semiconductor as large as 1.5 × 10^10^ cm^−3^; *E*_F−N_ is the Fermi level of the N-type Si substrate we used, which can be calculated with formula (1); *μ*_*n*_ is the electron mobility of the N-type silicon; and *σ* and *ρ* are the conductivity and resistivity of the N-type Si we used, respectively. The electron concentration of the N-type Si substrate used here can be calculated with formula (2). The conduction and valence band of N-type Si locate at 4.05 eV and 5.17 eV below the vacuum energy level, respectively. As shown in Figure 2(c), the Fermi level of the N-type Si is calculated as 4.16/4.27/4.32/4.38/4.46/4.52 eV under the different resistivity of 0.01/0.5/5/50/1000/10000 *Ω*·cm. The voltage responses of the dynamic N-Si/N-Si homojunction generator with resistivity of 0.01/0.5, 0.01/5, 0.01/50, 0.01/1000, and 0.01/10000 *Ω*·cm are 0.10, 0.15, 0.21, 0.30 and 0.35 V, respectively, indicating that the voltage output is positive related to the Fermi level difference as high as 0.11, 0.16, 0.22, 0.30 and 0.36 eV (Figure 2(d)).

The current of our dynamic NN Si homojunction generates with voltage output synchronously, which is totally different from the alternating current output of the triboelectric nanogenerator, which is based on the displacement current in the Maxwell equation and always outputs alternating current with the limit of the insulating dielectric materials. The continuous direct current of the dynamic NN Si homojunction generator confirms the above work mechanism, which can provide reference for other types of generators. In order to further prove that the Fermi level difference and built-in field play a vital status in the electrical generation, N-type silicon wafer was dragged along with other N-type silicon wafers with the same Fermi levels, as shown in Figure 2(e). The result of the performance of two same P-type silicon wafers with the same Fermi levels is shown in Figure S3. It is found that no stable direct voltage can be produced compared with the NN Si homojunction with different Fermi levels, indicating the importance of Fermi level difference, as shown in Figure 2(f). There are no built-in electric fields and rebounding diffusion carriers generated in the interface, so no continuous direct current could be generated. It is noteworthy that some noise signals are generated with the mechanical movement, which is caused by the thermal exciton movement without the dimensional separation of carriers under the interfacial *E*.

**Figure 3:**
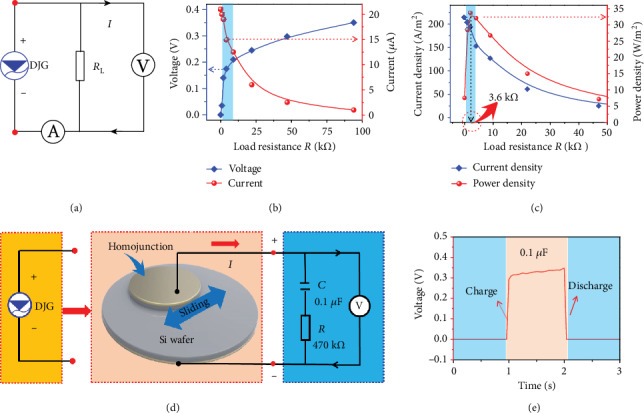
The electrical properties and potential practical application of the dynamic homojunction generator. (a) The circuit diagram of the operating circuit under the load *R*. (b) Voltage and current output of dynamic NN homojunction as a function of electrical load *R*. (c) Current density and power density of dynamic NN homojunction as a function of electrical load *R*. (d) The circuit diagram of charging a capacitor *C* (0.1 *μ*F) with the dynamic Si NN homojunction generator. No additional rectification circuit has been used. (e) The real-time voltage of the capacitor *C* (0.1 *μ*F), which is charged with the dynamic NN homojunction generator continuously.

To measure the power output and its internal impedance of our dynamic homojunction generator, the electrical output under different electrical load *R*_L_ has been explored with the measurement circuit in Figure 3(a). As shown in Figure 3(b), the work voltage is largely enhanced but the work current is decreasing with the increase of *R*_L_, as a result of the loss of internal resistance *R*_S_. Accordingly, the current density and power density output under different *R*_L_ have also been explored, as shown in Figure 3(c). As driven by the semiconductor-based dynamic homojunction, the working circuit consisted of an internal resistance *R*_s_, a load resistance *R*_L_, and a junction capacitor. When *R*_L_ comes to 3.6 k*Ω*, a peak power output density of 33.6 W/m^2^ has been achieved, which is equal to *R*_s_ of the dynamic NN Si homojunction device. It is noteworthy that the internal impedance of this semiconductor homojunction-based direct current generator is rather low (~k*Ω*) compared with the impedance of polymer material-based nanogenerators (~M*Ω*), which is matching with the impedance of the semiconductor-based information electronic device (~k*Ω*), indicating its potential in the common electronic device energy supply.

To develop its potential applications in the internet of things, a dynamic NN Si homojunction generator is used to charge a capacitor of 0.1 *μ*F without any rectification circuit. As shown in the detailed circuit diagram of Figure 3(d), a dynamic homojunction generator (DJG), a large capacitor of 0.1 *μ*F, a series resistance of 470 k*Ω*, and a voltage multimeter are used to construct a capacitor charging circuit. The detailed optical picture of the capacitor charging circuit is shown in Figure S4, in which the charging capacitor is measured with a Keithley 2010 system. A voltage output larger than 0.3 V was achieved under the continuous movement process of the dynamic NN Si homojunction generator, as shown in Figure 3(e). In particular, no external rectification circuit was added here and the charging speed is ultrahigh as a result of the high current density output of the dynamic NN Si homojunction generator (Figure S5). Therefore, this simple dynamic NN Si homojunction generator is a potential candidate for light and miniaturized in situ energy generator, which can work as a simple and feasible mechanical energy harvesting device to convert low-frequency disordered mechanical motion into electricity.

**Figure 4:**
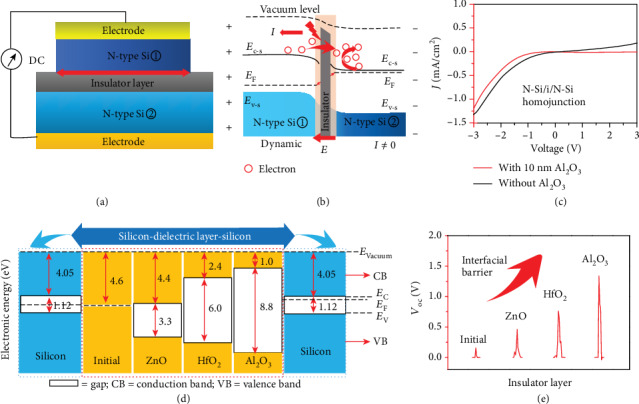
Enhanced voltage of the dynamic homojunction generator based on the N-i-N structure. (a) Schematic illustration of the dynamic N-Si/insulator/N-Si junction. (b) Band diagram and carrier dynamic process of the dynamic N-Si/insulator/N-Si junction generator. (c) *J*‐*V* curves of the dynamic N-Si/N-Si junction generator with and without SiO_2_. (d) One-dimensional band alignment of the energy band structure for silicon with various dielectric layers (ZnO, HfO_2_, and Al_2_O_3_). (e) The increased *V*_oc_ of the dynamic N-Si/dielectric/N-Si junction generator with different Interfacial barrier heights.

As the generation of the current and voltage is caused by the rebounding carriers in the interface of homojunction, the voltage and current must be influenced by the barrier height of the interfacial barrier. To further explore the role of the barrier height of dynamic homojunction and develop a system semiconductor theoretical framework in these dynamic junction generators, different kinds of ultrathin dielectric layers are inserted into the dynamic NN Si homojunction for further experiments, as shown in Figure 4(a). As shown in the band diagram of dynamic N-Si/insulator/N-Si junction (Figure 4(b)), insulator layer with a large bandgap could act as a stable barrier between NN Si homojunction. Under the ultrahigh interfacial *E* from N-Si to other N-Si substrates with lower Fermi levels, the space electrons will transfer to the N-type Si with higher Fermi levels and drifting holes will transfer to the N-type Si with lower Fermi levels through the insulator layer, which is equivalent to a discharge process of the N-i-N junction capacitor. The barrier height of the NN homojunction has been largely increased with the inserted insulator layer. As shown in Figure 4(c), the current leakage under negative bias voltage has been largely decreased under the effect of the inserted Al_2_O_3_ layer, indicating the increased barrier height and enhanced threshold voltage. When we move the N-Si wafer along with the Al_2_O_3_ layer, more hot electrons with high energy can be generated and drove by the ultrahigh interfacial *E*, generating a higher voltage signal. As shown in the one-dimensional band alignment of Figure 4(d), the energy band structure of silicon with different dielectric materials (such as ZnO, HfO_2_, and Al_2_O_3_) is compared [67, 68]. The voltage output of these dynamic homojunction generators after inserting dielectric layers (ZnO, HfO_2_, and Al_2_O_3_) is 0.5/0.8/1.3 V (Figure 4(e)), respectively, among which the Si/Al_2_O_3_/Si structure behaves the highest voltage output and barrier height. According to the relationship between the band structure relativities and voltage output, the carriers are driven by the interfacial *E* and the voltage output is positive related to the barrier height.

## 3. Conclusion

In summary, we have proposed a direct current generator with high current density based on the dynamic homojunction through the dynamically mechanical movement between two same kinds of semiconductors with different Fermi levels, eliminating the role of triboelectric effect in the semiconductor-based generator. The physical mechanism is attributing to the rebounding effect of hot carriers by the ultrahigh interfacial *E*, origin from the breaking symmetry of generating and disappearing depletion region carriers in the dynamic homojunction, which may provide reference for other types of generators. In particular, as a majority carrier device, a dynamic NN Si homojunction direct current generator with short-circuit current density of 214.0 A/m^2^, open-circuit voltage of 0.35 V, and power density of 33.6 W/m^2^ can be achieved. Moreover, the internal impedance of this semiconductor homojunction-based direct current generator is rather low (~k*Ω*), which is matching with the impedance of the semiconductor-based electron component (~k*Ω*). Through applying the N-i-N structure, output voltage can be further improved to 1.3 V. Compared with other generators, this dynamic NN homojunction generator can convert mechanical energy into continuous direct current electricity in ultrahigh current density without the rectifying circuit and storage unit, which indicates its potential promising applications in many fields, such as portable wearable devices. This approach provides a simple and feasible way of converting low-frequency disordered mechanical motion into electricity, especially the biomechanical energy, wind power, and tidal energy.

## 4. Materials and Methods

### 4.1. Preparation of Silicon Wafers

A single polished silicon wafer with different doping types and concentrations was used in this work. Firstly, the SiO_2_ layer in the interface of the Si substrate was removed by being dipped into 10 wt% HF for 5 minutes. Then, the DI water was used for three times to remove the residual HF. To fabricate an ohmic contact electrode of silicon, 10 nm Ti and 100 nm Au were successively grown on the bake side of single polished silicon with the thermal evaporation method. A natural NN homojunction was achieved by simply pressing the N-type Si wafer closely on the other N-type Si wafer by a constant force. Furthermore, the ZnO/HfO_2_/Al_2_O_3_ layer with thickness of 10 nm was fabricated with the atomic layer deposition method on N-type Si wafer.

### 4.2. Physical Measurement

The rectification characteristic of the NN homojunction was recorded with a Keithley 2400 instrument and Agilent B1500A system. The electrical output signals were measured with the Keithley 2010 multimeter, which was recorded by a LabView-controlled data acquisition system with the sampling rate of 25 s^−1^. To control the parallel movement of semiconductor wafers, a control system was customized. Semiconductors were fixed on the desk and pressure sensor, respectively. Under the control of a microcontroller unit and computer, the pressure sensor moved horizontally along with the sliding rail at a constant speed. The pressure and speed can slightly change with a variation of 10% as the inherent mechanical error such as the flatness of the mechanical arm and frame.

## Supplementary Materials

Supplementary Materials Figure S1: picture and performance of dynamic PN junction generator. Figure S2: the work mechanism of P-Si/N-Si homojunction. Figure S3: the performance of two same P-type silicon wafers with the same Fermi level. Figure S4: the capacitor charging experiment. Figure S5: the real-time voltage of the capacitor charging experiment.
