# Supplementary material for: Interfacial Built-In Electric Field-Driven Direct Current Generator Based on Dynamic Silicon Homojunction
Source: Research (Wash D C). 2020 Jun 16;2020:5714754. doi: 10.34133/2020/5714754 (PMC7315393; doi:10.34133/2020/5714754)
Supplement: Supplementary Materials — Figure S1: picture and performance of dynamic PN junction generator. Figure S2: the work mechanism of P-Si/N-Si homojunction. Figure S3: the performance of two same P-type silicon wafers with the same Fermi level. Figure S4: the capacitor charging experiment. Figure S5: the real-time voltage of the capacitor charging experiment. [file 5714754.f1.docx]

**Type: Research Article**

**Interfacial Built-in Electric Field Driven Direct-Current Generator Based on Dynamic Silicon Homojunction**

*Yanghua Lu*^1^*, Qiuyue Gao*^1^*, Xutao Yu*^1^*, Haonan Zheng*^1^*, Runjiang Shen*^1^*, Zhenzhen Hao*^1^*, Yanfei Yan*^1^*, Panpan Zhang*^1^*, Yu Wen*^2^*, Guiting Yang^3^ and Shisheng Lin^1,4,*^*

^1^College of microelectronics, College of Information Science and Electronic Engineering, Zhejiang University, Hangzhou, 310027, P. R. China

^2^Wuxi Branch of Jiangsu Province Special Equipment Safety Supervision and Inspection Institute, Wuxi, 214071, P. R. China

^3^State Key Laboratory of space power technology, Shanghai Institute of Space Power Sources, Shanghai, 200245, P. R. China

^4^State Key Laboratory of Modern Optical Instrumentation, Zhejiang University, Hangzhou, 310027, P. R. China

^*^Correspondence: [shishenglin@zju.edu.cn](mailto:shishenglin@zju.edu.cn).

Supplementary Figures:

**1. Figure S1**


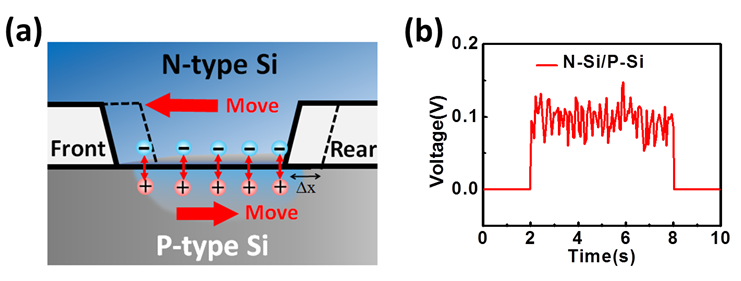


**Figure S1: picture and performance of dynamic PN junction generator.** (a) The schematic diagram of the dynamic N-Si/P-Si junction generator. (b) The voltage response of dynamic P-Si/N-Si junction generator under the continuous movement mode with a 5.0 N force and a speed of 10.0 cm/s.

**2. Figure S2**


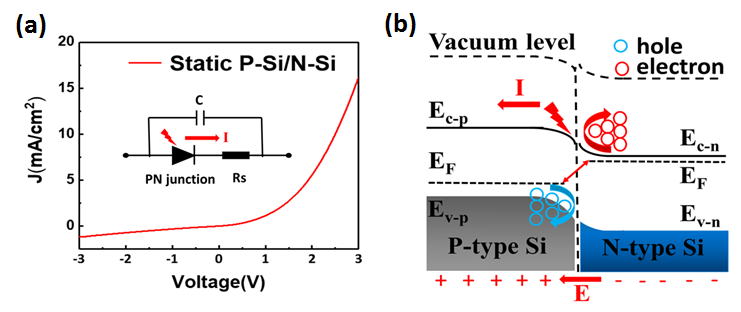


**Figure S2: the work mechanism of P-Si/N-Si homojunction.** (a) The rectification characteristic of P-Si/N-Si homojunction. Insert: The circuit diagram. (b) The band diagram of the dynamic silicon PN homojunction.

**3. Figure S3**


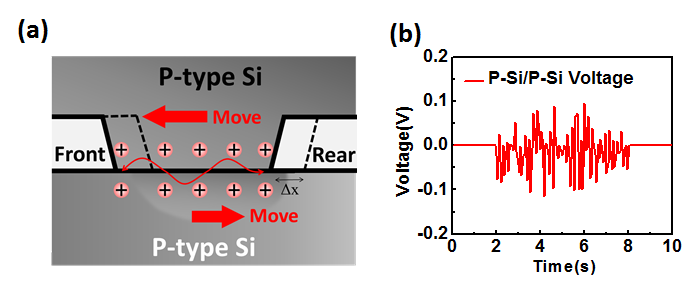


**Figure S3: the performance of two same P-type silicon wafers with same Fermi level.** (a) The schematic diagram of the dynamic P-Si/P-Si homojunction. (b) The voltage output of the dynamic P-Si/P-Si homojunction under the continuous movement.

**4. Figure S4**


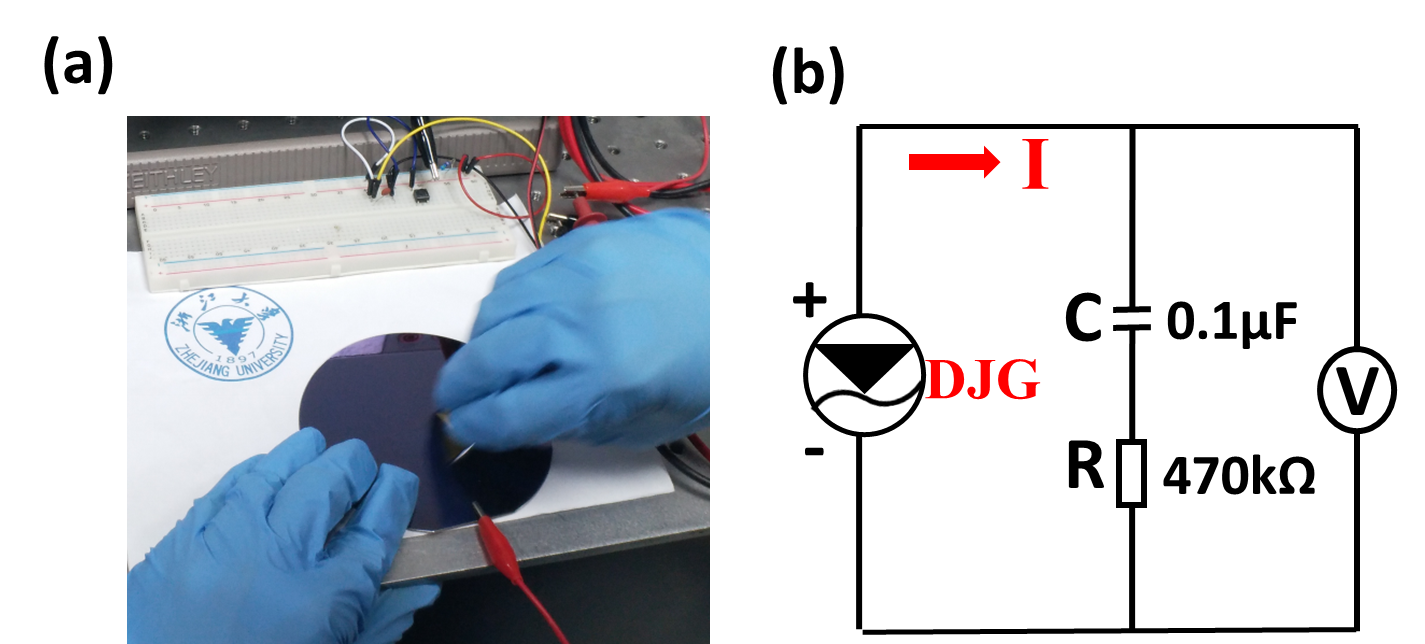


**Figure S4: the capacitor charging experiment.** (a) The captured optical picture of the capacitor charging experiment, which is measured with Keithley 2010 system. (b) The circuit diagram of charging a capacitor C (0.1μF) with the dynamic Si NN homojunction generator. No additional rectification circuit has been used.

**5. Figure S5**


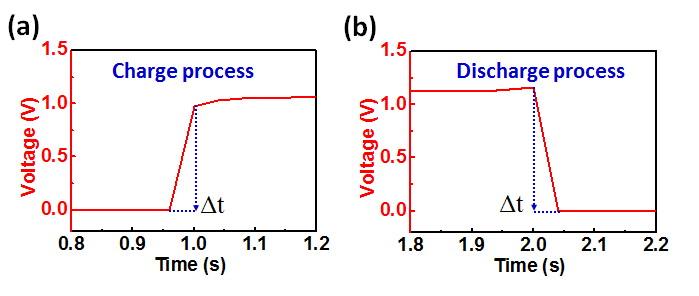


**Figure S5: the real-time voltage of the capacitor charging experiment.** (a) The real-time voltage of the capacitor C (0.1μF), which is changed with the dynamic Si NN homojunction generator. (b) The real-time voltage of the discharging capacitor C (0.1μF).
